# Electric hand warmer versus observation to avoid discomfort during scalp cooling for chemotherapy-induced alopecia prevention: a randomized study

**DOI:** 10.1038/s41598-023-46840-3

**Published:** 2023-11-09

**Authors:** Luciana Castro Garcia Landeiro, Diego Lopes Paim Miranda, Roberto Mathias Machado, Rodrigo Dienstmann, Matheus Costa e Silva, Ceci Figuerêdo da Silva, Adriana Lisbôa Ramalho de Castro, Ana Paula Teixeira dos Santos, Victor Hugo Valença Bomfim, Bruno Teixeira Machado, Michele Viviane Carvalho Rodrigues Gonçalves, Andréa Freitas Muniz Teixeira, Maira Jamile Santiago Costa, Priscila de Oliveira Dantas Viana, Pâmela Almeida, Clarissa Maria de Cerqueira Mathias

**Affiliations:** 1 Grupo Oncoclínicas- Bahia, Av. Milton Santos, 123 - Ondina, Salvador, BA 40170-110 Brazil; 2 Complexo Hospitalar Universitário Professor Edgard Santos (C-HUPES) - Universidade Federal da Bahia (UFBA), Savador, BA Brazil; 3 Escola Bahiana de Medicina e Saúde Pública (EBMSP), Salvador, BA Brazil; 4 OC Precision Medicine - Grupo Oncoclínicas, São Paulo, SP Brazil; 5 Faculdade de Medicina da Bahia, Universidade Federal da Bahia (UFBA), Salvador, BA Brazil; 6 Clínica AMO - Assistência Multidisciplinar em Oncologia, Salvador, BA Brazil

**Keywords:** Cancer, Oncology

## Abstract

Chemotherapy-induced alopecia (CIA) is a challenge in the management of cancer patients. Scalp cooling (SC) leads to reduction in CIA, however it is associated with significant adverse events, leading to 3–13% discontinuation rates. This pilot study evaluated the role of Electric Hand Warmers (EHW) on thermal (TC), sensorial (SCo) and general comfort (GC) in patients with breast cancer (BC) undergoing chemotherapy and SC to reduce CIA. Patients were randomly assigned to EHW use or observation. TC, SCo and GC were evaluated after each chemotherapy infusion. Favorable outcomes in both TC and SCo defined a positive result on GC. We analysed the impact of age, alopecia, chemotherapy regimen and EHW use in the different comfort scales using a Logistic Regression (LR) model. Forty women with early breast cancer were randomly assigned to EHW (n = 20) or observation (n = 20) during neo(adjuvant) chemotherapy. Median age was 53 years. In the EHW arm, favorable thermal response was reported by 79% versus 50% in the control arm (odds ratio [OR] 3.79, *p* < 0.001). SCo was satisfactory in 82% in the EHW arm versus 74% in the control arm (OR 1.62, *p* = 0.1). Overall, 73% in the EHW arm had favorable GC versus 44% in the control arm (OR 3.4, *p* < 0.001). Age, alopecia, and chemotherapy regimen did not impact on comfort measures. Conclusion: Our study suggests that the use of an EHW has a consistent favorable impact on TC and GC of BC patients under SC technology to prevent CIA.

## Introduction

Alopecia holds the potential to substantially diminish the quality of life (QoL) and negatively affect body image in patients with BC, many of whom consider hair loss to be the most traumatic aspect of chemotherapy treatment^1,2^. This fear can negatively affect outcomes, as up to 8% of patients with BC decline chemotherapy to avoid hair loss^3^. While chemotherapy-induced alopecia (CIA) is commonly viewed as transient, there exist rare instances where it manifests as a lasting condition^4^ Addressing and preventing alopecia can significantly enhance the QOL of patients undergoing chemotherapy, potentially fostering a willingness to embrace therapeutic interventions that offer survival advantages.

To prevent or reduce alopecia, a series of medical devices has been developed and tested. Most of these are based on scalp cooling systems that reduce the uptake of chemotherapeutic drugs in the hair follicles through a cutaneous vasoconstriction in the scalp^5^. The two scalp cooling devices mainly used worldwide are the DigniCap® System (Lund, Sweden) and the Paxman® System (Huddersfield, UK)^6^ These systems use a cap that is attached to a small, computer-controlled refrigeration machine that circulates cool liquid in the cap and controls the patient’s individual scalp temperature before, during, and after chemotherapy infusion ^7^ Cold temperatures also reduce cellular metabolic activity, which lessens the effects of cytotoxic chemotherapeutic agents on follicular cells^8–10^.

The prevalent side effects of scalp cooling encompass headaches, sensation of being chilled, and scalp pain, contributing to discontinuation rates ranging from 3 to 13%^11,12^. Additional adverse effects encompass pruritus as well as mild discomfort in the head, neck, or shoulder regions^11^. Also, strategies like wetting hair before scalp cooling can enhance intolerance and increase discontinuation rates^13^.

Hand Warmers, which are found in disposable (recrystallization and platinum catalysts) or electric formats, are used to produce heat and to warm cold hands^9^. Initially designed to generate heat for warming cold hands, hand warmers have found frequent application in outdoor activities, and on medical field to mitigate symptoms of Raynaud’s disease^14^ Disposable hand warmers can last from 30 min to 24 h, while Electric Hand Warmers (EHW) are rechargeable, and allow more precise temperature control, in addition to providing greater security regarding continuous maintenance, without oscillations^15,16^. So far, there are no reports of toxicity with the use of the EHW.

Our hypothesis posited that the implementation of EHW could enhance the well-being of patients undergoing scalp cooling to mitigate Chemotherapy-Induced Alopecia (CIA).

## Materials and methods

### Participants

The study included women aged 18 years or older, with an ECOG performance status of 0–1, diagnosed with stage I–III invasive breast cancer. Patients were scheduled to commence neoadjuvant or adjuvant chemotherapy, in conjunction with the Paxman Scalp Cooling System (Paxman Coolers Limited, Huddersfield, UK), to prevent or reduce CIA.

The chemotherapy regimens encompassed a spectrum of options including dose dense or conventional Doxorubicin and Cyclophosphamide (ACdd or AC), followed by weekly or dose dense Paclitaxel (wP or Tdd); Docetaxel and Cyclophosphamide (TC); Paclitaxel and Trastuzumab (TH); Docetaxel, Carboplatin, Trastuzumab, and Pertuzumab (TCHP); and the combination of AC-T with Trastuzumab.

Regarding laboratory parameters, individuals were deemed ineligible if their hemoglobin levels were less than 10 g/dL, white blood cell counts were below 1.5 × 10^3^/L, or platelet counts were lower than 100 × 10^3^/L. Further exclusion criteria encompassed a recent history of malignancy within the last five years (excluding basal cell carcinoma of the skin or cervical carcinoma in-situ), pregnancy, presence of other severe medical conditions, including clinically notable heart disease, liver and kidney failure, and any prior experience of chemotherapy with scalp cooling prior to study enrollment.

### Devices and procedure

The EnergyFlux Ellipse, an electric rechargeable device manufactured by Human Creations, a brand under KindMinds Innovations Inc. from Taiwan, served as the chosen EHW (Energy-Generating Hand Warmer) for this study. This device stood out due to its dual temperature settings: 42 °C (low) and 48 °C (high). The size of the device fits in a palm^17^.

Participants were instructed to hold a device in each hand throughout their time seated in the chemotherapy infusion chair. The cooling process began at the lower temperature, but participants had the option to escalate to the higher temperature with the assistance of a nurse. At the conclusion of the scalp cooling session, participants were prompted to report any discomfort or issues associated with the device. They were also asked to indicate if they had stopped using the device and, if so, the duration: less than 30 min, 30–60 min, or more than 60 min.

Upon completion of each chemotherapy participants were asked to provide written feedback on both thermal and sensory comfort. Responses were collected using a 5-point Likert scale, encompassing: (a) Thermal Comfort: Very Warm, Warm, Neutral, Cold, and Very Cold (with Neutral or Warm indicating favorable thermal comfort); (b) Sensory Comfort: Very Comfortable, Comfortable, Neutral, Uncomfortable, and Very Uncomfortable (with Comfortable and Very Comfortable indicating favorable sensory comfort).

Ultimately, positive outcomes in both thermal and sensory comfort categories determined an overall positive assessment of general comfort.

### Sample size calculation and statistical analysis

This study was designed to achieve 80% power to detect 35% improvement in patients' thermal comfort response, with a margin of error of 5%. In total, 20 patients were required per study arm, considering a margin of 15% lost to follow-up or non-evaluable for the primary endpoint.

We evaluated the impact of age (≤ or > 50 years), alopecia (grade 0 or 1/2), chemotherapy regimen (with or without taxanes), and EHW use (yes or no) in the different comfort scales using Fisher’s Exact Test. Due to the difference in number of cycles that each patient could receive, we considered each patient questionnaire during the chemotherapy session as an individual sample and compiled them to compare the two arms. Statistical analyzes were evaluated using R Statistical Programming Language version 4.1.2^18^.

### Ethical approval

This study was performed in line with the principles of the Declaration of Helsinki. Approval was granted by the Comitê de Ética em Pesquisa do Hospital Santo Antônio/Obras Sociais Irmã Dulce, CAAE nº 80,305,017.0.0000.0047.

### Consent to participate

Informed consent was obtained from all individual participants included in the study.

## Results

Between April 2018 and October 2018, a total of 40 patients were enrolled in this trial. The discontinuation rate for scalp cooling was 20% in the intervention group and 25% in the control group. The reason for discontinuation among all participants was grade 2 alopecia. Discomfort with the scalp cooling system was not reported as the primary reason for discontinuing its use by any of the participants.

The sociodemographic characteristics of the patients are comprehensively detailed in Table 1. The median age of the participants was 53 years, with 27 individuals (67.5%) having completed higher education and 20 patients (50%) presenting comorbidities. Furthermore, the predominant chemotherapy regimen utilized was dose-dense ACdd-T (55%). Most patients were diagnosed with stage II disease (55%), primarily categorized as HR + HER2- (67.5%), followed by triple-negative cases (22.5%), and HER2 + cases (10%).

**Table 1:** Sociodemographic variables of the study patients.

Sociodemographic variables	Control group % (N = 20)	Intervention group % (N = 20)
Age at diagnosis	53.75* (SD 11.92)	53.15* (SD 13.35)
Escolarity (completed college/university)	75 (n = 15)	60 (n = 12)
Comorbidities (yes)	40 (n = 8)	60 (n = 12)
Scalp cooling discontinuation rate^#^	25 (n = 5)	20 (n = 4)
Chemotherapy		
Neoadjuvant	55 (n = 11)	55 (n = 11)
Adjuvant	45(n = 9)	45 (n = 9)
Chemotherapy protocol		
ACDD-TDD	60 (n = 12)	50 (n = 10)
TC	15 (n = 3)	25 (n = 5)
AC Every 3 weeks → weekly TAXOL	15 (n = 3)	15 (n = 3)
TCHP	5 (n = 1)	5 (n = 1)
TH	5 (n = 1)	5 (n = 1)
Anti-HER2 Therapy (NO)	50 (n = 10)	90 (n = 18)

*Mean (SD 12.82).

^#^All patients who discontinued the EWH did so because they had alopecia grade 2, and decided not to use scalp cooling any longer.

**Figure 1:**
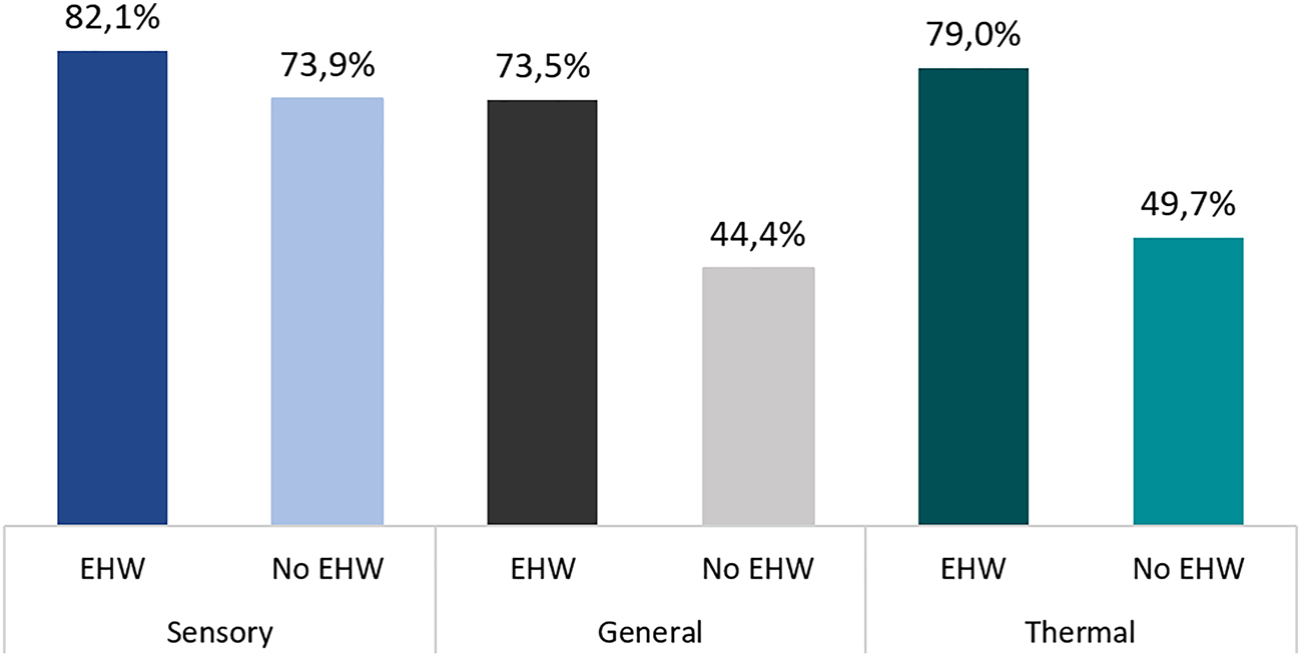
Proportion responses regarding thermal comfort, sensory comfort, and general comfort.

**Figure 2:**
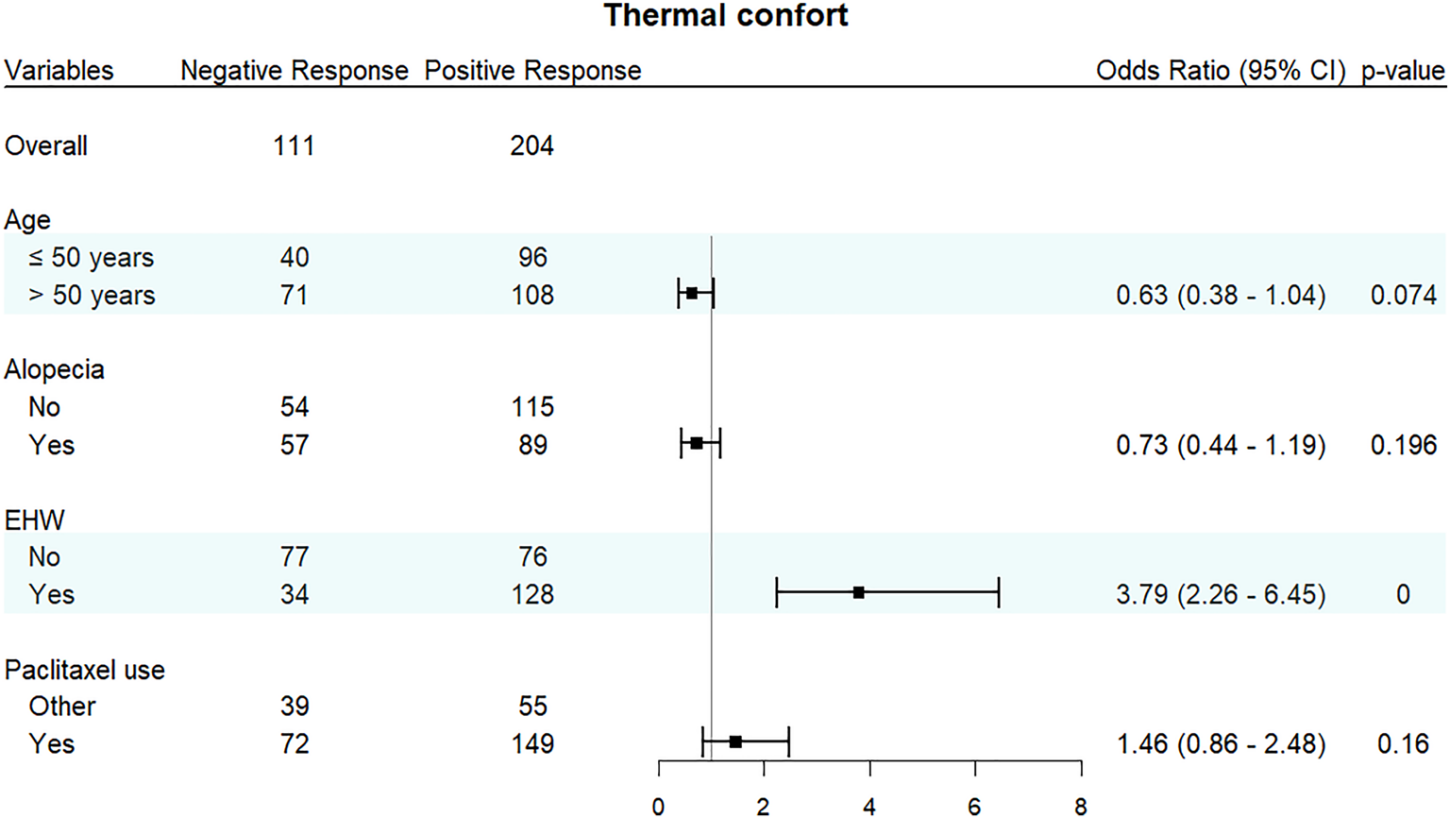
Measures of thermal comfort by age, alopecia, use of EHW, and use of paclitaxel.

Patients in the EHW group demonstrated a notably heightened favorable thermal comfort response (79%), presenting a sharp contrast to those who were assigned to the control group (50%), as depicted in Fig. 1 and Fig. 2 (Odds Ratio 3.79; 95% Confidence Interval 2.26 to 6.45; *p* < 0.001). Factors such as age, hair loss, and paclitaxel usage showed minimal impact on thermal comfort, as illustrated in Fig. 2.

**Figure 3:**
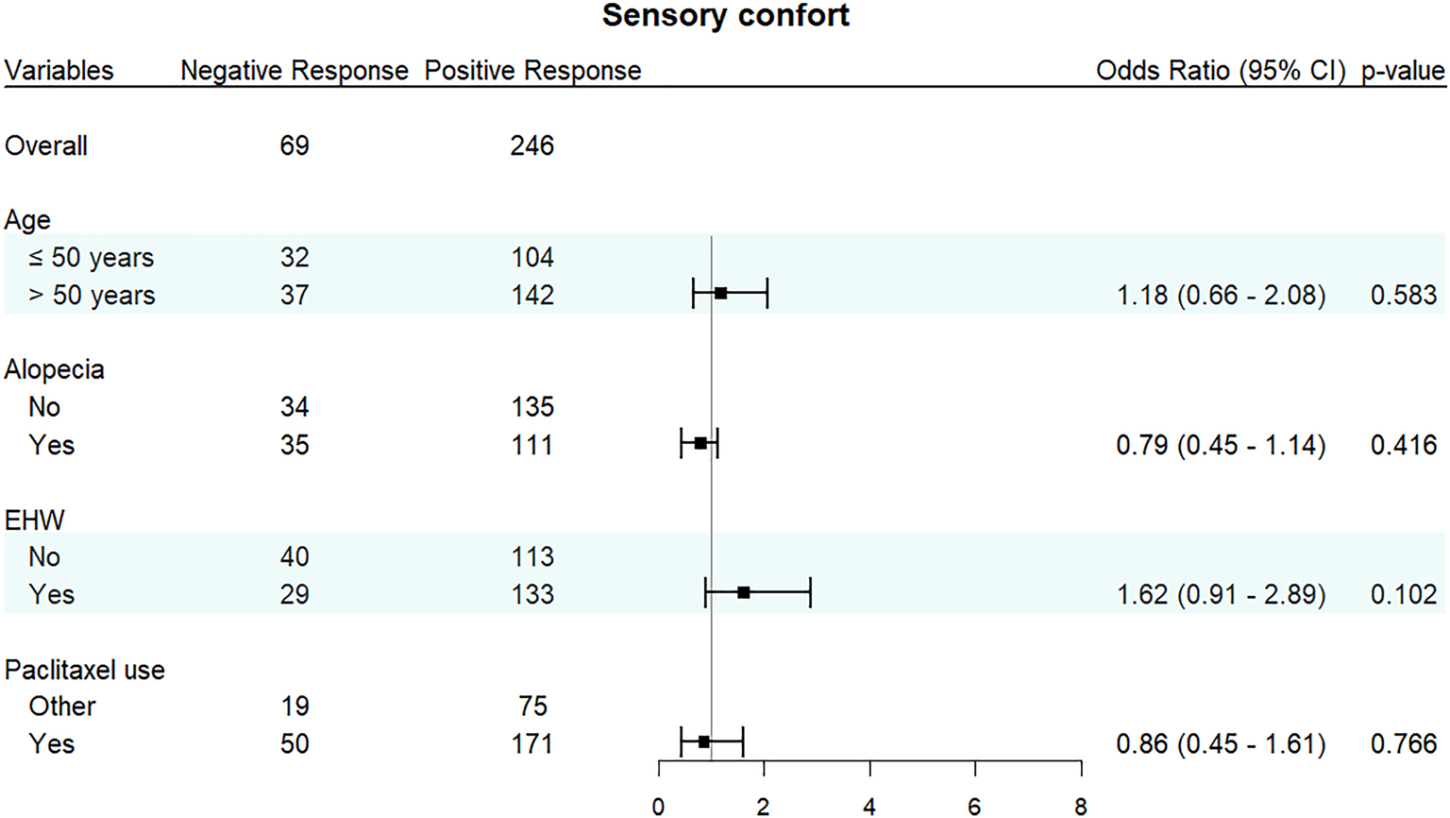
Sensory comfort measures by age, alopecia, use of EHW, and use of paclitaxel.

The association between EHW utilization and improved sensory comfort did not achieve statistical significance when compared to the control group (Odds Ratio 1.62; 95% Confidence Interval 0.91 to 2.89; *p* = 0.102). A closer examination indicates that 82% of patients in the EHW arm reported satisfactory comfort, while 74% did so in the control group (shown in Fig. 1). Likewise, age, alopecia, and the application of paclitaxel exhibited no significant correlation with sensory comfort, as demonstrated in Fig. 3.

**Figure 4:**
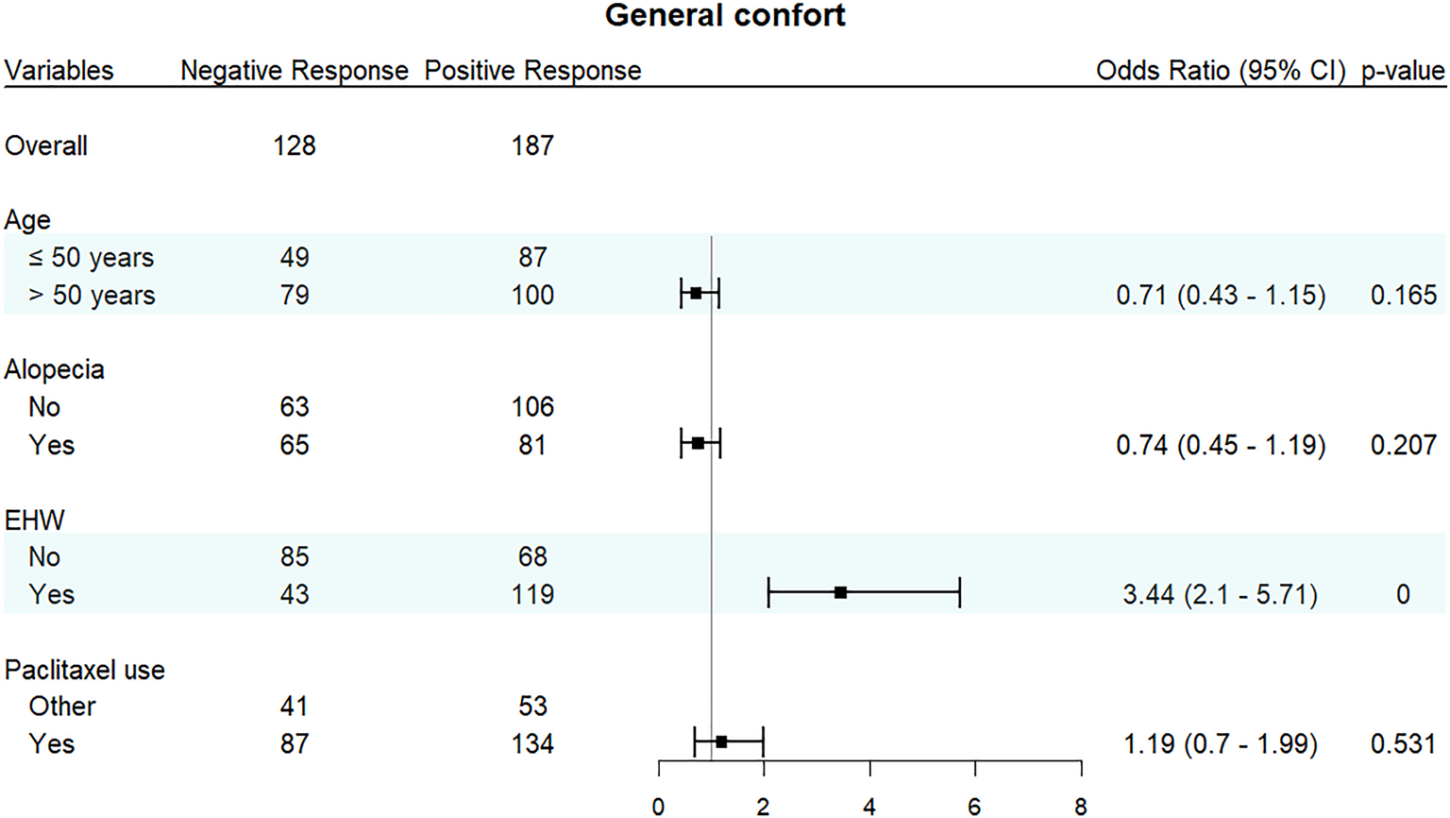
General comfort measures by age, alopecia, use of EHW, and use of paclitaxel.

Patients who utilized the EHW demonstrated a notably superior response to overall comfort compared to individuals in the non-EHW group (Odds Ratio 3.44; 95% Confidence Interval 2.1 to 5.71; *p* < 0.001), as visually illustrated in Fig. 2. As evident from Fig. 1, an impressive 73% of patients in the EHW arm reported a positive general comfort response, while only 44% did so within the control arm. Notably, factors such as age, alopecia, and paclitaxel usage showed no significant influence on the overall comfort experienced by patients, as depicted in Fig. 4.

Throughout the course of this study, no patients chose to increase the temperature. Each participant consistently took brief breaks from the device, lasting less than 30 min, primarily for restroom visits. None of the patients reported experiencing any sensation of heat discomfort or described adverse events related to the use of the EHW. Importantly, no patient dropped out because of heat intolerance.

## Discussion

Alopecia stands as one of the most dreaded side effects experienced by women undergoing chemotherapy. As many as 8% of female patients opt out of breast cancer chemotherapy treatment due to the distressing prospect of hair loss^3^. The scalp cooling technique has demonstrated the capacity to diminish the likelihood of total hair loss by up to 42%, establishing its status as a highly favored method^10,17–22^.

However, two out of every three individuals report experiencing a degree of discomfort during scalp cooling, and discontinuation of the technique is a relevant issue. We postulated that the incorporation of EHW would provide heightened comfort for patients undergoing chemotherapy with scalp cooling. Nonetheless, our pilot study's findings underscore that the primary determinant of adherence continues to be the effectiveness of the scalp cooling approach. This effectiveness can be subject to different variables, including hair type and previous hair treatments^23,24^. All patients that stopped using scalp cooling, in the present study, reported stopping because of grade 2 alopecia.

In the present study, the utilization of EHWs has been shown to be feasible and is linked with encouraging improvements in well-being, potentially leading to a reduction in rates of discontinuation.

Overall, patients exhibited a significant increase in terms of general comfort (59%) and thermal comfort (66%) when utilizing EHW (both with *p* < 0.001), while the impact on sensory comfort due to EHW usage was not statistically significant.

This study faces limitations primarily stemming from its relatively small sample size and a significant discontinuation rate observed among participants, with 20% discontinuation in the intervention group and 25% in the control group. Our initial expectation had been a 15% attrition rate due to loss to follow-up or non-evaluability concerning the primary endpoint. Nevertheless, it is worth noting that this attrition rate might have been mitigated to some extent. This was because, in light of the variation in the number of chemotherapy cycles received by individual patients across different chemotherapy protocols included in the study, we treated each patient questionnaire completed during a chemotherapy session as an individual data point. This approach allowed us to aggregate these data points and perform meaningful comparisons between the two study arms. Recent studies have shown early discontinuation rates of up to 28%, so it is of utmost importance to carefully consider these rates when planning future investigations^20^.

It is also important to highlight the predominance of younger patients (median age of 53 years old), which restricts our ability to fully assess the effect of EHW on the comfort of elderly patients. Some data suggests that this populations tends to be more sensitive to cold and less sensitive to heat^25^.

Another limitation arises from the uncertainty surrounding the potential impact of EHW on the development of neuropathy. The incidence of neuropathy in the different groups was not evaluated in this small study. Notably, a counterintuitive strategy for managing the risk of chemotherapy-induced peripheral neuropathy (CIPN) involves the use of ice-cold gloves/socks, posing a paradoxical contrast to the current EHW approach^26^. In this context, a follow-up study should prioritize the inclusion of patients undergoing therapies associated with a minimal risk of neuropathy and/or consider incorporating these therapies as a stratification criterion.

It's important to note that the absence of blinding in this study's design is primarily because the intervention involves a thermal device, making it impractical to blind the participants using such a device.

The findings of this study lend support to the hypothesis that incorporating EHW could yield beneficial outcomes as a complementary therapy alongside scalp cooling techniques. Future studies should explore the impact of EHW on patients' adherence to scalp cooling, their tolerance, as well as enhancements in patient satisfaction and overall quality of life during chemotherapy. Additionally, examining potential adverse events linked to EHW within a broader patient population is crucial for a comprehensive understanding.

## Data Availability

The dataset generated during and/or analyzed during the current study are not publicly available due to participant privacy but are available from the corresponding author on reasonable request.

## References

[1] Choi EK (2014). Impact of chemotherapy-induced alopecia distress on body image, psychosocial well-being, and depression in breast cancer patients. Psychooncology.

[2] Kim IR (2012). Perception, attitudes, preparedness and experience of chemotherapy-induced alopecia among breast cancer patients: A qualitative study. Asian Pac. J. Cancer Prev..

[3] Macquart-Moulin G, Viens P, Bouscary ML (1997). Discordance between physicians’ estimations and breast cancer patients’ self-assessment of side-effects of chemotherapy: An issue for quality of care. Br. J. Cancer.

[4] Kang D, Kim I-R, Choi E-K (2019). Permanent chemotherapy-induced alopecia in patients with breast cancer: A 3-year prospective cohort study. Oncologist.

[5] Rossi A, Caro G, Fortuna MC, Pigliacelli F, D’Arino A, Carlesimo M (2020). Prevention and treatment of chemotherapy-induced alopecia. Dermatol. Pract. Concept..

[6] Silva GB, Ciccolini K, Donati A, Hurk CVD (2020). Scalp cooling to prevent chemotherapy-induced alopecia. An. Bras. Dermatol..

[7] Kruse M, Abraham J (2018). Management of Chemotherapy-Induced Alopecia with Scalp Cooling. J. Oncol. Pract..

[8] Hurk CJGVD, Mols F, Vingerhoets AJJM, Breed WPM (2010). Impact of alopecia and scalp cooling on the well-being of breast cancer patients. Psycho-oncology.

[9] Hesketh PJ (2004). Chemotherapy-induced alopecia: Psychosocial impact and therapeutic approaches. Support Care Cancer..

[10] Auvinen PK (2010). The effectiveness of a scalp-cooling cap in preventing chemotherapy-induced alopecia. Tumori.

[11] Rugo HS (2015). Clinical performance of the DigniCap system, a scalp hypothermia system, in preventing chemotherapy-induced alopecia. ASCO Meeting Abstr..

[12] Kadakia KC, Rozell SA, Butala AA, Loprinzi CL (2014). Supportive cryotherapy: a review from head to toe. J. Pain Symptom Manag..

[13] Heibloem RE, Komen MMC, Ilozumba OU (2023). Minimal added value of wetting hair before scalp cooling to prevent chemotherapy-induced alopecia in cancer patients—Results from the Dutch Scalp Cooling Registry. Support Care Cancer.

[14] Sands WA, Kimmel WL, Wurtz BR, Stone MH, McNeal JR (2009). Comparison of commercially available disposable chemical hand and foot warmers. Wilderness Environ. Med..

[15] Coffman JD (2000). Raynaud's phenomenon. Curr. Treat Options Cardiovasc. Med..

[16] Mascoli, G. Warmer hands (and toes) through chemistry. *ScienceIQ Blog Network* https://www.scienceiq.com/Facts/WarmerHands.cfm

[17] Human creations. EnergyFlux Ellipse Series 5200mAh Rechargeable Hand Warmer / External Battery[Internet]. [place unknown: publisher unknown]; 2019 [updated unknown; cited 2023 Oct 17th]. Available from: https://www.human-creations.com/product-page/energyflux-ellipse-5200mah-rechargeable-wrap-around-hand-warmer-usb-external-b

[18] R Core Team. R: A language and environment for statistical computing. R Foundation for Statistical Computing, Vienna, Austria. URL https://www.R-project.org/ (2021).

[19] Shen XF, Ru LX, Yao XB (2021). Efficacy of scalp cooling for prevention of chemotherapy induced alopecia: a systematic review and meta-analysis. Eur. Rev. Med. Pharmacol. Sci..

[20] Carbognin L, Accetta C, Giorgio D (2022). Prospective study investigating the efficacy and safety of a scalp cooling device for the prevention of alopecia in women undergoing (neo)adjuvant chemotherapy for breast cancer. Curr. Oncol..

[21] Silva GB, Ciccolini K, Donati A, Hurk CVD (2020). Scalp cooling to prevent chemotherapy-induced alopecia. An. Bras. Dermatol..

[22] Bajpai J (2020). Randomised controlled trial of scalp cooling for the prevention of chemotherapy induced alopecia. Breast.

[23] Araoye EF, Stearns V, Aguh C (2020). considerations for the use of scalp cooling devices in black patients. J. Clin. Oncol..

[24] Dilawari A (2021). Does scalp cooling have the same efficacy in black patients receiving chemotherapy for breast cancer?. Oncologist.

[25] Panet MDF, Araújo VMD, Araújo EHSD (2022). Thermal sensation index for elderly people living in Brazil. Int. J. Biometeorol..

[26] Shigematsu H, Kimura Y, Itagaki T, Yasui D (2023). Persistent weekly paclitaxel-induced peripheral neuropathy in early breast cancer patients enrolled in a randomized trial of cryotherapy. Medicine (Baltimore)..

